# Spatial compatibility interference effects: a double dissociation between two measures

**DOI:** 10.1080/13506285.2015.1110653

**Published:** 2015-12-11

**Authors:** Alexander J. Kirkham, Steven P. Tipper

**Affiliations:** ^a^ Department of Psychology, University of York, YorkYO10 5DD, UK

**Keywords:** Spatial compatibility, Simon effect, double-dissociation, spatial interference, EMG

## Abstract

In spatial compatibility tasks, when the spatial location of a stimulus is irrelevant it nevertheless interferes when a response is required in a different spatial location. For example, response with a left key-press is slowed when the stimulus is presented to the right as compared to the left side of a computer screen. However, in some conditions this interference effect is not detected in reaction time (RT) measures. It is typically assumed that the lack of effect means the irrelevant spatial code was not analysed or that the information rapidly decayed before response. However, we show that even in conditions where there appears to be no spatial interference when measuring RTs, effects can nevertheless be detected after response when recording facial electromyography responses. This dissociation between two measures highlights the importance of diverging methods to investigate visuomotor processes as conclusions based on only one measure can be misleading.

To successfully interact with complex environments requires that action should be computed rapidly in an efficient and automatic manner. However a problem with systems where actions can be computed automatically is that competing actions can be activated in parallel, impairing processing whilst selection of the appropriate action is undertaken. A classic example of this selection problem is the Simon effect (e.g., Simon & Rudell, 1967). In the basic task participants report the colour of a stimulus with a left or right key-press. For example, if red press left key, if green press right key. The stimulus to be identified can be randomly presented to the left and right sides of space. The basic result, observed many times, is that responses are faster when response and spatial location of the stimulus are compatible (response left / stimulus presented to the left) than when they are incompatible (response left / stimulus presented to the right).

This effect shows that although stimulus location is irrelevant to the task it is automatically computed and linked to response output systems. That the incorrect spatial response is activated on incompatible trials is confirmed in studies recording the lateralized readiness potential (LRP) where the incorrect spatial response is briefly activated while the correct response is emerging (e.g., Leuthold & Sommer, 1993).

Hommel (1994) reviewed the Simon effect noting that a key property of the spatial response competition was that the irrelevant to-be-ignored response property rapidly decayed. For example, Simon, Acosta, Mewaldt, and Speidel (1976) required participants to delay response until a go signal was presented. As this delay increased up to 350 ms the spatial response competition declined. Similarly, slowing down the processing of the target stimulus by manipulating stimulus quality also reduced spatial interference effects (Hommel, 1993). For example, comparing an easily discriminated clear-red vs. clear-blue with a difficult discrimination of reddish vs. bluish increased overall RTs and reduced Simon interference (Hommel, 1994). Hence the decline in interference from irrelevant spatial information is the result of a spontaneous rapid decline in activation.

Aside from time, other properties of a stimulus can reduce the interference effects of irrelevant properties. For example, in Stroop colour word conflict tasks, placing the to-be-ignored word in a separate spatial location to the target colour reduces interference (Kahneman & Henik, 1981) as does increasing spatial distance between locations (Gatti & Egeth, 1978; see also Roelofs & Lamers, 2007). In other studies, whether the irrelevant property is within or between objects, has confirmed that interference can be extinguished (Driver & Tipper, 1989).

A key issue is how the removal of interference effects is interpreted. For example, it is usually assumed that when there is no longer any evidence for interference effects from irrelevant stimulus properties in reaction time (RT) measures, they are no longer encoded. However, the lack of an effect may not be due to lack of encoding, and other measures might in fact reveal that the irrelevant stimulus property was indeed processed and is influencing behaviours other than speed of response.

At this point we need to be clear about the initial motives of this study. It was *not* designed to specifically investigate situations where competition is no longer detected. The apparent double dissociation between RT measures and EMG activation to be reported here was a serendipitous discovery. The study was in fact initially designed to examine how visuomotor fluency could influence subsequent ratings of the characteristics of a person, and how embodied emotional reactions evoked by response competition, as measured by EMG, influenced these processes.

Previous work has shown that perceptual fluency/effort (e.g., Zajonc, 1980) and motor fluency/effort (e.g., Hayes, Paul, Beuger, & Tipper, 2008) can influence emotion, and that implicit emotional responses can be detected via EMG recording of the muscles associated with expressed emotion (e.g., Winkielman, Schwarz, Fazendeiro, & Reber, 2003). In the current study we manipulated response conflict, as previous work has shown that brain regions such as the anterior cingulate cortex (ACC) encode both response conflict and negative emotional reactions to conflict (Barbas & Pandya, 1989; Holland & Gallagher, 2004; O'Doherty, 2004). However, the stimulus discrimination task in the current study was much harder, with RTs close to 200 ms slower than in previous work (e.g., Tipper & Bach, 2008) resulting in no spatial interference effects in one condition, and hence the task was not suitable for our initial goals.

However, the experiment did reveal previously unknown properties of spatial compatibility effects. To preview our findings of a double dissociation between RT and EMG measures of spatial competition, we again confirm that in some circumstances interference from irrelevant spatial information is no longer observed. That is, there are no detectable differences between compatible and incompatible trials when measuring RT. And yet, we also discovered that in the latter situation where there are no effects of competing spatial information in RT measures, there is nevertheless discrimination of compatible and incompatible spatial information in the EMG activation up to multiple-seconds after a response has been completed.

## Methods

### Participants

A total of 27 female students aged between 18 and 24 years (*M* = 21.11 years, *SD* = 2.35) participated in the study in return for course credit or a payment of £6. All participants had normal or corrected-to-normal vision.

### Materials and apparatus

**Figure 1.**
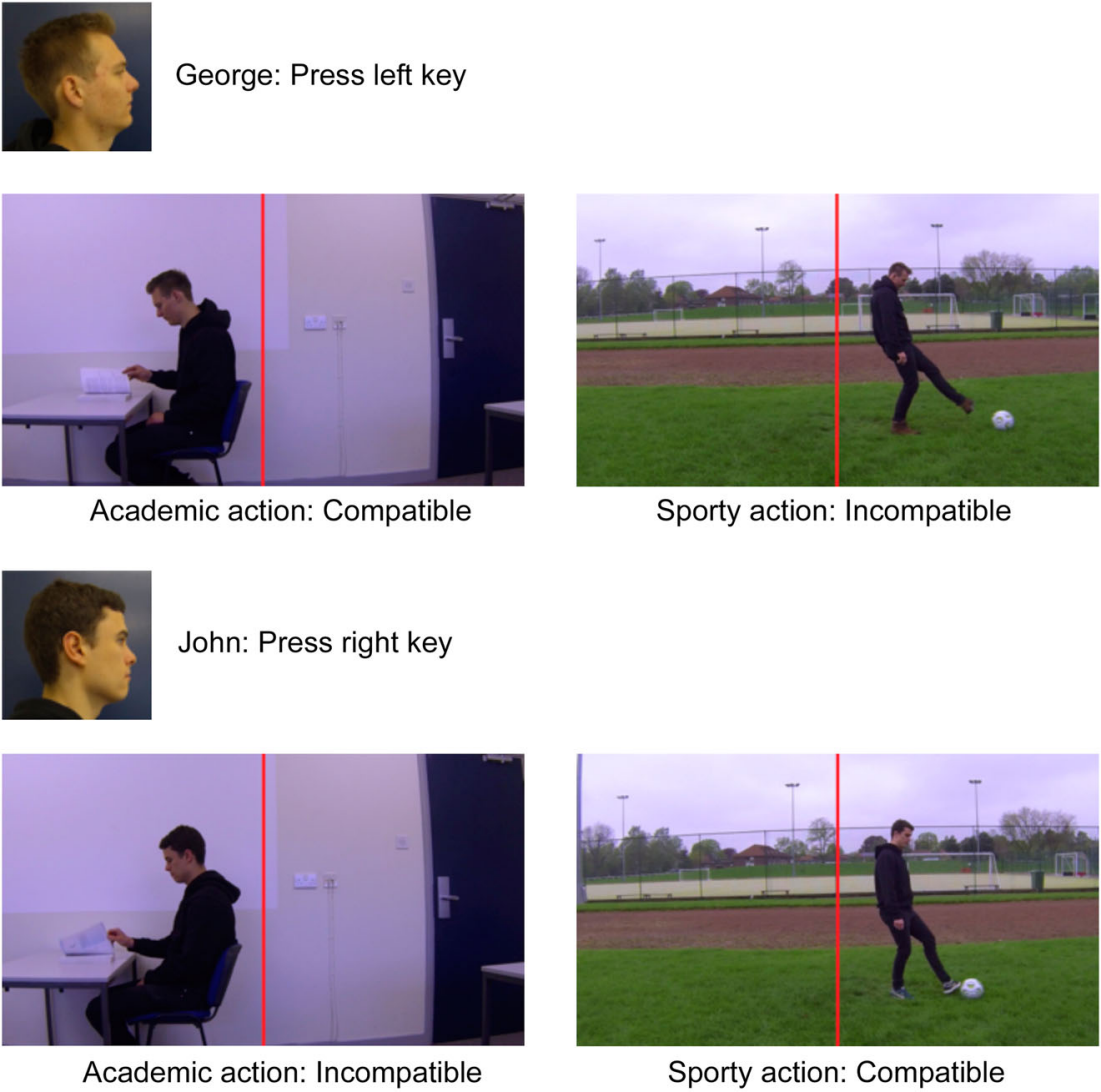
Examples of trial stimuli. Note that the visual angle of the head in the Academic scene was substantially larger (3.8° × 2.9°) than in the Sporty scene (1.9° × 1.4°), facilitating person identification in the former. The red vertical line indicates the centre of the screen, and was not presented to participants.

Participants were seated at the experimental PC at a distance of approximately 60 cm from the screen. The study was presented using E-Prime 2 and controlled using a standard QWERTY keyboard. The stimuli used in the study consisted of eight videos of two actors performing two actions in two directions. Each actor was filmed kicking a football, and separately opening a book. Each video was filmed in a contextually correct scenario (kicking a football outside on grass by a sports pitch; opening a book at a desk). Each video lasted 3000 ms in total, beginning at the start of the action and finishing at the completion of the action. Four videos were filmed and adjusted so as the head of each actor was shown on the left side of the frame-centre (the centre of the frame to the first point of contact with each actor's head measured: Kicking = 3.1° degrees; Book = 4.1°), with the action continuing in a leftward direction towards the edge of the screen. These videos were then mirrored to enable rightward facing videos to be generated. This resulted in the final eight stimulus videos. Participants were assigned to one of four experimental variations, permitting counterbalancing to be performed according to both actor response key (Q and P) and side of performed action on-screen (the left side showing an Academic action, and the right side a sporty action, or vice-versa. See Figure 1).

### Design and procedure

All participants were presented with standardized task instructions on-screen in conjunction with a verbal discussion of the task instructions to ensure full understanding. Each participant saw four of the eight videos, all of which were presented an equal number of times in a random order. Of these videos, two showed each actor (George and John) kicking a football (Sporty action) on *one side of the screen* (the left), whilst the other two videos showed each actor opening a book (Academic action) on *the other side of the screen* (the right). For half of the participants this configuration of kicking to the left and opening a book to the right was shown, whilst for the remaining half the presentations were reversed so as kicking was to the right and opening a book was to the left. The participant was directed to make a key-press response declaring which actor was performing the action, regardless of what the action was. Accordingly, the key-press response keys were also counterbalanced across participants. For half of the participants identification of George required pressing the Q key, whilst identification of John required pressing the P key. For the remaining half of participants the key-press response keys were reversed.

Consider Figure 1 that shows examples of the stimuli and main conditions. Participants were required to identify George with a left key-press and John with a right key-press. Note that when viewed opening a book George is to the left of the screen and is facing left, and hence is compatible with the left key-press, unlike when he is on the right and facing right while kicking a ball. In contrast, John has the opposite stimulus-response compatibility assignment, in the Sporty scene on the right and facing right compatible with the right key-press, and on the left and facing left in the incompatible Academic scene.

**Figure 2.**
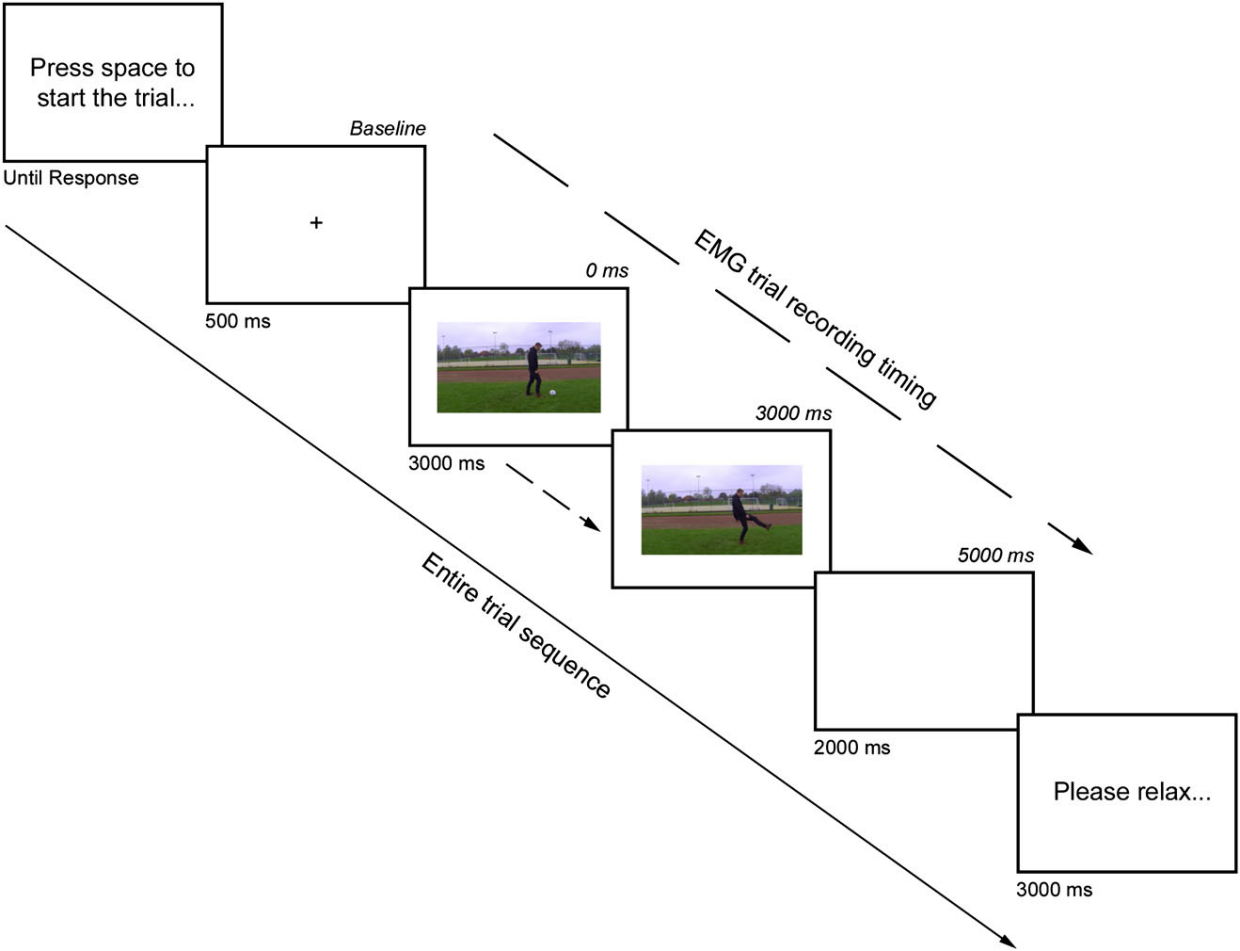
A typical trial within the study. Separate arrows detail both the entire trial sequence timing, but also the timeframe during which EMG signals were recorded.

An initial block of 16 practice trials was performed to ensure that participants were comfortable with the task. There then followed an experimental block of 160 trials. Each trial was self-initiated by the participant pressing the spacebar. A fixation cross was presented for 500 ms before the video was shown for 3000 ms. Following this a blank screen was shown for 2000 ms, accompanied by an error tone only if they selected the incorrect actor with a key-press. Finally a 3000 ms interval was presented asking the participant to relax before the following trial (see Figure 2).

### EMG apparatus and methodology

EMG provides the benefits that it can be non-invasive and is sensitive, thereby being able to detect responses under the visual detection threshold (van Boxtel, 2010). Different patterns of EMG activity correlate with various indexes of emotion and effort. The contraction of the corrugator is a crucial action unit for the expression of anger and distress (Ekman & Friesen, 1978). When presenting affective pictures corrugator activity increases linearly as ratings become more negative and decreases as ratings become more positive (Lang, Greenwald, Bradley, & Hamm, 1993; Larsen, Norris, & Cacioppo, 2003), and it is also correlated with amygdala responses to affective pictures (Heller, Greischar, Honor, Anderle, & Davidson, 2011; Heller, Lapate, Mayer, & Davidson, 2014), a brain region that is associated with emotion. Therefore we predict that corrugator activity will be larger on incongruent trials, reflecting the negative emotion evoked from response conflict. Predictions for the zygomaticus are less clear. Although previous work showed greater zygomaticus response on compatible trials (Cannon, Hayes, & Tipper, 2010) the stimuli and procedures were quite different and effects might not generalize. Furthermore, other work demonstrates this muscle shows a bivalent response profile, correlating with affective ratings of both positive and negative stimuli (Lang et al., 1993; Larsen et al., 2003; Wu, van Dijk, & Clark, 2015) and may reflect cognitive effort.

Facial electromyographic (EMG) activity was measured from the zygomaticus major and corrugator supercilii muscles at a resolution of 2000 Hz using a BioPac MP150 system paired with EMG100C modules. Two pairs of 4 mm Ag/AgCl electrodes filled with conductive electrolyte gel were secured upon the left-hand side of the face of each participant using adhesive discs. The electrodes were sited according to the guidance of van Boxtel (2010). A ground electrode was also placed upon the forehead.

Following the completion of each recording, the raw signal from each muscle was filtered using a bandpass filter (20 Hz–500 Hz) and a notch filter of 50 Hz, before being rectified and smoothed with an integration window of 50 ms.

EMG activity was measured across each trial as a percentage ratio between mean muscle activity during the 500 ms of the fixation screen (to be treated as a baseline), and subsequent 500 ms time windows. EMG activity was recorded for 5000 ms following the fixation period of each trial. This ensured that for each trial there was a clear muscle activity time-course.

All participants were naive as to the true application of the EMG system, instead being told that researchers were investigating frontal lobe activity. No participant was aware, or became aware, that facial muscle activity was being recorded.

### Participant and trial rejections

Due to the sensitivity of EMG measures it was necessary to remove some trials and, as a result, five participants from further analyses. Data from these participants must be excluded due to the gross skewing that would occur through retaining the data. Rejected trials were selected where it was evident that substantial and unrelated participant movements had occurred (e.g., sneezing, coughing or other movements); this was performed by the researcher who was blind to the trial condition. Such movements are exacerbated due to participants being unaware that facial EMG is being recorded; therefore on occasion they may make no effort to avoid excessive movements. Data from the remaining participants had < 10% of trials removed overall due to these described movements.

## Results

### Reaction times

**Figure 3.**
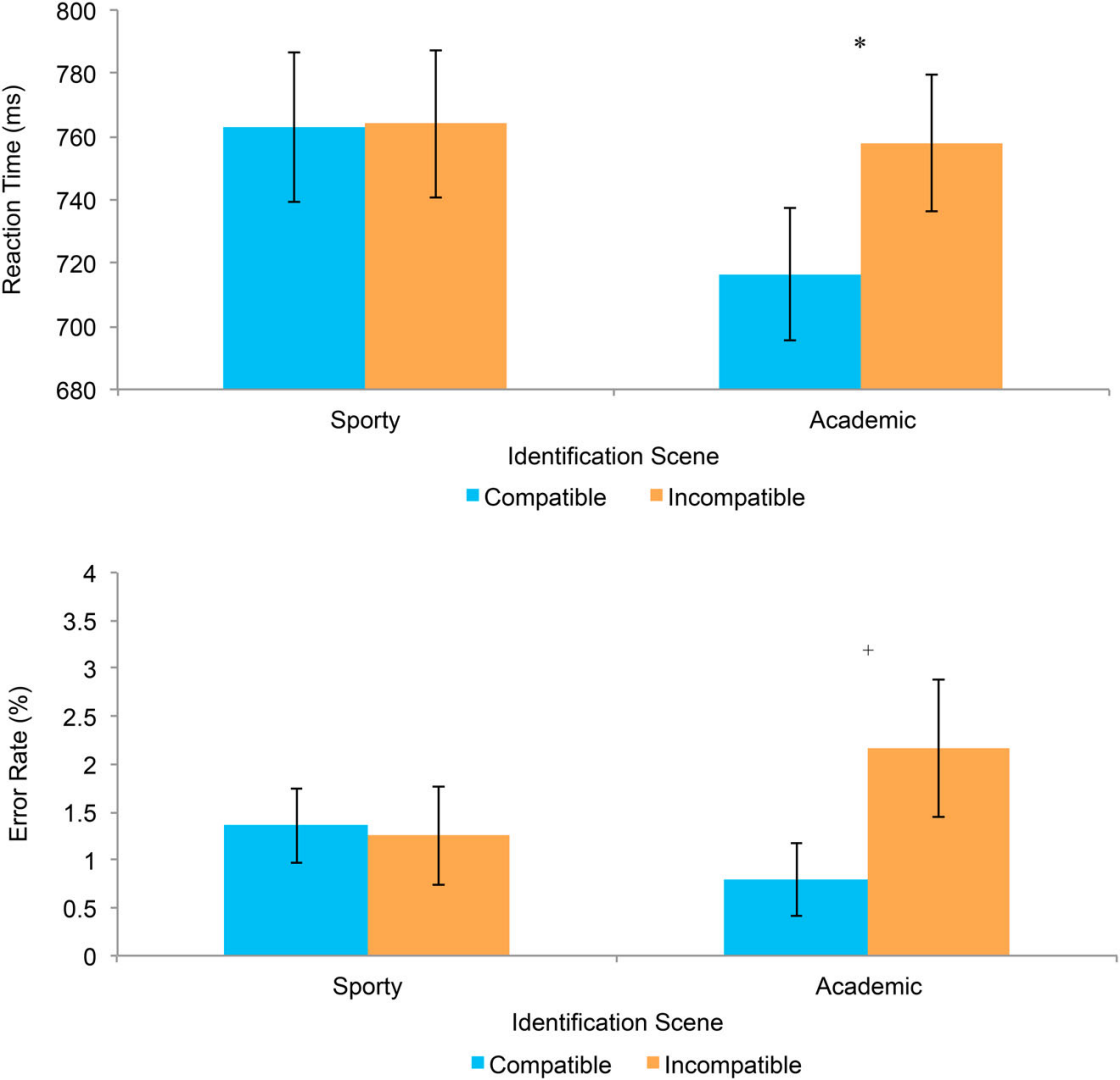
Shows the reaction time effects and error data rates (collapsed across actors) for both the Academic and Sporty video scenes, comparing both compatible and incompatible responses. Error bars denote standard error values. *p = .01; ^+^ *p* *=* .1.

An initial analysis revealed no main effect of Person/Actor, therefore further analyses were collapsed across Person and analysed in a two-way ANOVA with the factors Action and Compatibility where reaction times to the identification of each actor in each scene was measured according to whether the spatial response was compatible or incompatible. A main effect of Action was obtained [*F*(1,21) = 12.77, *p* = .002, 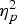 = .38] where faster identifications were made during the Academic trials than during the Sporty trials. A main effect of Compatibility was also obtained [*F*(1,21) = 5.16, *p* = .034, 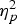 = .20] with faster responses during compatible trials than incompatible trials. Furthermore an interaction of Action and Compatibility was observed [*F*(1,21) = 5.32, *p* = .031, 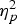 = .20] where the compatibility effect was only significant in the Academic scene [*t*(21) = 2.85, *p* = .010] and not in the Sporty scene [*t*(21) = 0.11, *p* = .91] (see Figure 3).

These data confirm the previous findings concerning spatial compatibility effects in Simon-like tasks. That is, reaction times to identify the two individuals when observed in the Sporty scene were significantly longer than when they were viewed in the Academic scene. This impaired performance was due to the reduced visual angle of the face in the Sporty scene (1.9° × 1.4°) compared to the Academic scene (3.8° × 2.9°). This slowed processing was accompanied by reduced spatial interference effects, as predicted by models that propose rapid decay of competing irrelevant spatial codes.

**Figure 4.**
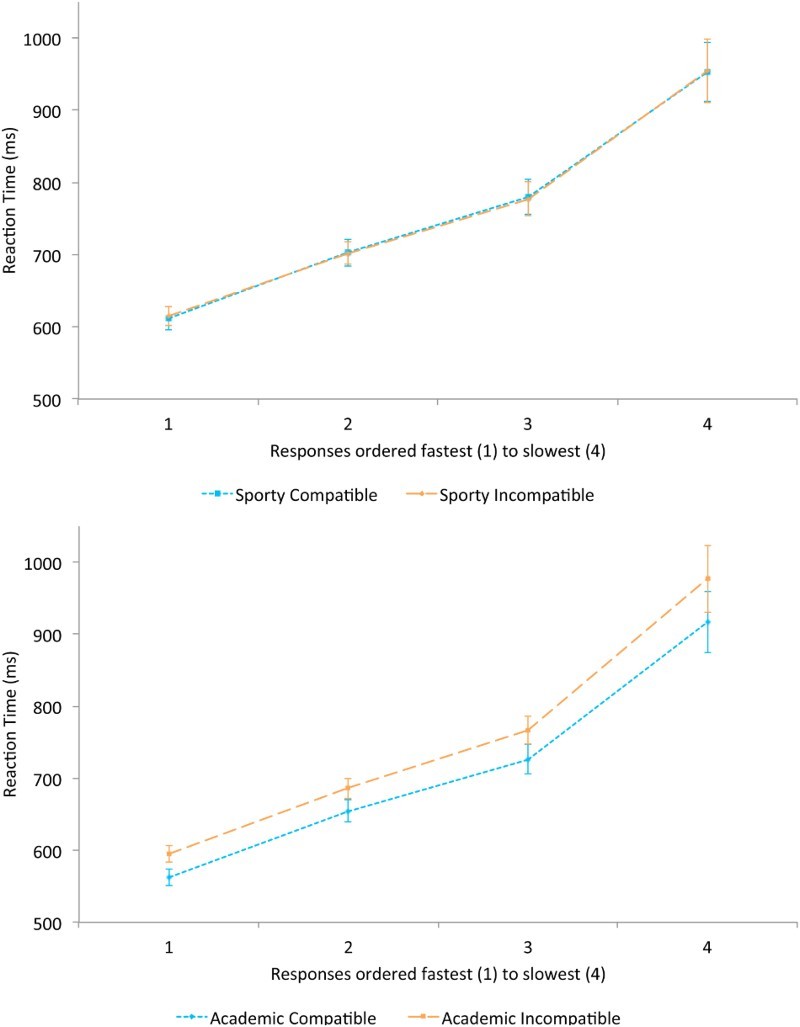
Sporty scenes (top) and Academic scenes (below). Line graphs showing the RT (in ms) for compatible and incompatible trials, ranked from fastest to slowest by individual subject.

However, although RTs were significantly longer in the Sporty scene condition, further analysis was necessary to confirm that the loss of interference effects was due to longer RTs. The data was divided in to 4 RT bins enabling analysis of interference effects through the RT distribution. A clear prediction is that interference should be greater in the faster RTs. A three-way ANOVA analysed factors of Action (Sporty/Academic), Compatibility (compatible/incompatible) and Bin (4 RT bins ranked from fastest to slowest responses in quartiles). Main effects of Action [*F*(1,21) = 12.66, *p* = .002, 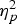 = .38] and Compatibility [*F*(1,21) = 5.39, *p* = .030, 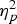 = .20] were obtained where both Academic and compatible trials produced the fastest responses. A main effect of Bin also occurred [*F*(3,63) = 97.09, *p* < .001, 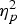 = .82]. Again the interaction of Action and Compatibility produced a significant outcome [*F*(1,21) = 5.71, *p* = .026, 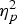 = .21] as a result of significant response compatibility effects only in the Academic condition. There were however no significant interactions of Action and Bin [*F*(3,63) = 1.87, *p* = .14, 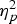 = .08], Compatibility and Bin [*F*(3,63) = 0.78, *p* = .51, 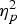 = .036], or Action, Compatibility and Bin [*F*(3,63) = 1.03, *p* = .38, 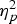 = .047] (see Figure 4).

### Error rates

Again there was no main effect of Person/Actor; therefore identical analyses were performed to those within the Reaction Times. The analysis revealed no main effect of Action [*F*(1,21) = 0.17, *p* = .68, 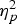 = .008], or Compatibility [*F*(1,21) = 1.42, *p* = .25, 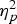 = .063]. There was no significant interaction of Action and Compatibility obtained [*F*(1,21) = 2.39, *p* = .14, 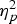 = .10].

Although the interaction was not significant, the general pattern of the error rates provides some support for the RT data findings. In particular, during the Academic scene condition there were more errors in the incompatible than the compatible conditions.

### Electromyography

All analyses presented here are separated by individual muscle, since there are often substantial differences in the reactivity of the zygomaticus (cheek) and corrugator (brow) muscles due to the differences in overall muscle size. Further, analyses are presented where EMG activity is measured as the ratio between the baseline period (500 ms of the fixation screen) and the following 4500 ms of the trial. Note that the final 500 ms of each trial is not analysed since this often contained large unrelated movements, as participants were aware of the upcoming trial. Analyses were conducted using a three-way repeated measure ANOVA with factors of Action (Academic scene and Sporty scene), Compatibility (compatible and incompatible response), and Time (nine time periods of 500 ms duration).

### Corrugator

No main effect of Action [*F*(1,21) = 0.24, *p* = .63, 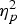 = .012] or Compatibility [*F*(1,21) = 2.18, *p* = .16, 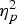 = .094] was obtained. A main effect of Time did however emerge [*F*(8,168) = 9.81, *p* = .001, 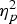 = .32]. The interaction of Action and Compatibility [*F*(1,21) = 4.26, *p* = .052, 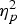 = .17] was marginally significant, and Action, Compatibility and Time [*F*(8,168) = 2.32, *p* = .044, 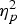 = .10] was significant. To better understand the nature of this data, analyses were conducted within each individual scene and comprised of a two-way ANOVA with the factors of Compatibility and Time.

**Figure 5.**
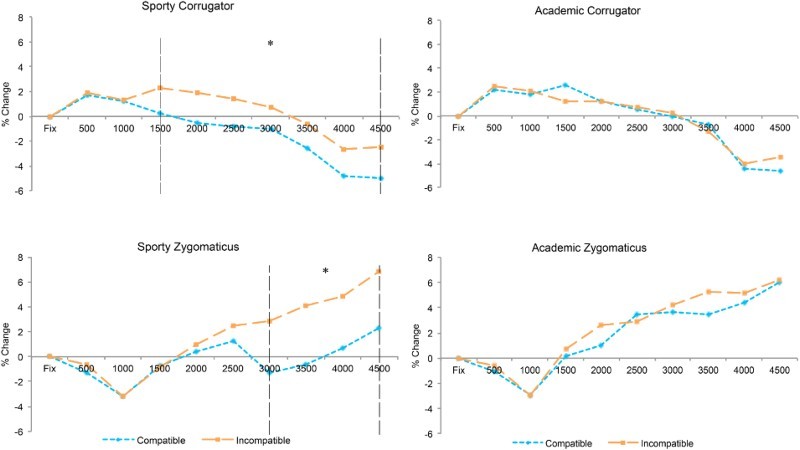
EMG activity as recorded from the corrugator and zygomaticus muscles according to the action being viewed and responded to. Dashed vertical lines indicate time-periods with significant differences between compatible and incompatible trials where *p < .05.

Within the Sporty scene, main effects of both Compatibility [*F*(1,21) = 7.77, *p* = .011, 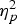 = .27] and Time [*F*(8,168) = 7.67, *p* = .001, 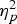 = .27] were obtained. Further, an interaction of Compatibility and Time occurred [*F*(8,168) = 2.54, *p* = .032, 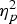 = .11], indicating that the activity toward incompatible trials was significantly greater than that toward compatible trials (see Figure 5). To further examine this interaction, paired *t*-tests were performed to assess for differences between compatible and incompatible trials within each time-period and found significant differences in all bins from 1500 ms onwards (all *p*s < .05, differences at 2000 and 2500 ms *p* < .01).

Within the Academic scene, no main effect of Compatibility [*F*(1,21) = .007, *p* = .93, 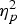 < .001] was obtained. Although a main effect of Time [*F*(8,168) = 9.12, *p* = .001, 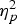 = .30] was significant, this did not result in a significant interaction of Compatibility and Time [*F*(8,168) = 1.25, *p* = .30, 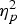 = .056] (see Figure 5). For clarity, identical analyses were performed within each time period as with the Sporty scene, but no significant differences were obtained.

### Zygomaticus

Within the three-way ANOVA, strong main effects of Action [*F*(1,21) = 7.68, *p* = .011, 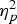 = .27], Compatibility [*F*(1,21) = 4.73, *p* = .041, 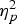 = .18] and Time [*F*(8,168) = 9.06, *p* < .001, 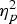 = .301] were obtained. There were no significant interactions of Action and Compatibility [*F*(1,21) = 1.77, *p* = .20, 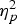 = .078] and Action, Compatibility and Time [*F*(8,168) = 1.69, *p* = .18, 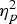 = .075].

However, to be consistent with the corrugator analysis above and motivated by the data patterns observed in Figure 5, a separate two-way ANOVA with the factors of Compatibility and Time for each action were undertaken. Within the Sporty scene, main effects of Compatibility [*F*(1,21) = 7.11, *p* = .014, 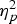 = .25] and Time [*F*(8,168) = 6.65, *p* = .002, 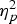 = .24] were obtained. Further, an interaction of Compatibility and Time emerged [*F*(8,168) = 3.19, *p* = .040, 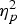 = .13], again suggesting that muscle activity during incompatible trials was significantly greater than that found during compatible trials (see Figure 5). As with the corrugator, paired *t*-tests were conducted to determine if there were significant differences between compatible and incompatible trials within each time period. Significant differences were found in all bins from 3000 ms onwards (all *p*s < .05, difference at 3000 ms *p* < .01).

In contrast, within the Academic scene no main effect of Compatibility [*F*(1,21) = 0.38, *p* = .55, 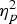 = .018] was obtained. A main effect of Time [*F*(8,168) = 8.06, *p* < .001, 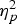 = .28] was obtained, but this did not affect the interaction of Compatibility and Time [*F*(8,168) = 0.49, *p* = .69, 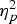 = .023] (see Figure 5). As with the Academic scene within the corrugator muscle, no significant differences were obtained comparing compatible and incompatible responses when assessing each time period.

## Discussion

This experiment has identified a double dissociation between two measures of spatial compatibility effects. We examined the standard measure of response competition, where RTs are slowed when a competing spatial code is activated. We also introduced a measure of response competition via EMG recordings of facial muscles. Previous work has shown that EMG correlates with various forms of emotion and effort evoked by fluency of stimulus processing. The contraction of the corrugator reflects the negative emotions; thus while viewing stimuli of different emotional valence its activity increases with increasingly negative stimuli (e.g., Larsen et al., 2003), and it correlates with activity of the amygdala when viewing emotional stimuli (e.g., Heller et al., 2014). Hence we predicted that the response competition evoked by the irrelevant spatial property of the stimulus would produce dis-fluent processing and negative emotional responses which would be revealed by greater corrugator activity during incompatible trials. However, although we expected the zygomaticus to also discriminate compatible and incompatible conditions, the direction of the effect was less predictable. The zygomaticus muscle has been shown to have a bivalent response profile, producing movements to both positive and negative stimuli (Lang et al., 1993; Larsen et al., 2003; Manssuer, Hayes, Pawling, & Tipper, in press), and on occasion being overall more responsive to negative than positive stimuli (Wu et al., 2015). Therefore, it was not clear whether it would be more active in the dis-fluent/effortful incompatible trials reflecting negative response to greater effort, or more active in the compatible fluent trials. And as noted, the zygomaticus is somewhat less robust than the corrugator response, perhaps reflecting competing processes.

Confirming previous findings (e.g., Hommel, 1994; Simon & Rudell, 1967), irrelevant spatial information that is incompatible with a response that has to be produced interferes with response output. For example, while making a response with the left hand a stimulus presented to the right side of space will slow down response relative to a stimulus presented on the same (left) side of response. Importantly this spatial response competition is only observed in the Academic scenes, there is no hint of any interference in the Sporty scenes. Although the lack of effect in the latter may be affected by the overall longer RTs to identify people in Sporty scenes, this is not a complete explanation as it is not supported by the time-course bin analysis. At this time we do not have a clear account of why spatial interference effects are not detected. One possibility is that the ball in the Sporty scene is very salient and attracts attention. This could produce a spatial object-based conflict that is opposite to the more global spatial frame. For example, when the person is on the left facing left, this is compatible with a left response. However, the person is to the *right* of the salient ball, producing the opposite compatibility (we thank a reviewer for this point). A further possibility resides in the added distractions of viewing more potent actions, as in the Sporty videos; clearly there is more activity on-going in comparison to the Academic videos, and the additional elements may cause distraction. However, note that there is no variation within the videos and therefore any novelty is likely to have diminished early on.

However, the main point is that compatibility/interference effects are very robust in the Academic scene, being observed throughout the full RT distribution. Note that the stability shown in the Academic condition is not unprecedented. Whilst such findings may not normally be expected in a typical horizontal Simon task, similar results have been obtained when stimuli are presented vertically rather than horizontally leading to the finding that the Simon effect decays over time in the horizontal format but crucially not in the vertical format (Vallesi, Mapelli, Schiff, Amodio, & Umiltà, 2005). Additionally, where there is a dual-task element to a Simon task, and hence competition for resources, the Simon effect can remain stable over the spectrum of RTs (Suarez, Vidal, Burle, & Casini, 2015). These effects are however never detected in the Sporty scene (see Figure 4). Does this mean that in the latter case the irrelevant spatial information is not represented as RT is unaffected by compatibility? This is clearly not the case, because when observing the EMG response to the compatible and incompatible trials, the opposite pattern is detected. That is, during the responses to the Academic displays where RT compatibility effects are observed, there is no evidence that EMG discriminates compatible from incompatible trials. In sharp contrast, in the Sporty trials where there is absolutely no evidence for compatibility effects in RTs, there is a late emerging discrimination of compatible from incompatible trials in the EMG. Hence a double-dissociation has been identified.

It has been noted before (e.g., Driver & Tipper, 1989; Wascher & Tipper, 2004) that lack of an RT interference effect from irrelevant distractors does not necessarily mean that the irrelevant stimulus was not encoded. Logicians term this fallacy as the denial of the antecedent as it assumes that any identification of the distractor necessarily interferes with response. In the Driver and Tipper study, non-interfering distractors nevertheless produced negative priming shortly after response was completed, and in the Wascher and Tipper study, no inhibition of return effects were detected in RT but EEG did discriminate between the critical cued and uncued conditions. Such findings confirm the encoding of the irrelevant stimuli even when there is no effect on behaviour. The current results are reminiscent of these earlier findings, in that an apparently non-interfering spatial stimulus property affects EMG activity after the response to the stimulus is completed.

The opportune finding that we would not have predicted was the lack of relationship between RT and EMG. When RTs revealed response compatibility effects in the Academic scene, the facial muscles did not discriminate between compatible and incompatible trials. This would suggest, counter to our proposals, that the dis-fluency in response did not evoke an emotional reaction, at least not one we could detect. However, in the Sporty condition where there was no hint of compatibility effects in RTs, EMG did reveal negative emotional reactions to incompatible trials.

Other studies have also noted similar relationships between behaviour (RTs) and emotion. For example, Fritz and Dreisbach (2013) found effects of response compatibility on subsequent emotional responses even when no overt response was required. Hence emotional encoding is not dependent on overt behaviour. Second, Wirth, Pfister, and Kunde (2015) have shown an asymmetry between RT measures of response competition and emotion. For example, increasing the probability of encountering negative emotional stimuli will reduce subsequent RT interference in a spatial Simon task, but the opposite relationship is not observed: increasing response competition does not effect emotional processing.

At this time we can only speculate as to the processes mediating these counter-intuitive effects. First, Otte, Habel, Schulte-Rüther, Konrad, and Koch (2011) report that response interference effects measured via EMG increase as EMG RTs increase, the opposite pattern to that observed with finger press RTs in Simon tasks. Second, Cannon et al. (2010) also noted slower emergence of effects with EMG in that the discrimination of compatible from incompatible trials appeared a few hundred milliseconds after response had been completed. Hence the EMG measure of emotional reaction to dis-fluency/effort is not necessarily detected during response selection and execution where dis-fluency can be observed in finger-press RTs, rather it seems to be a review process where emotion is evoked by incompatible trials after the response is completed.

A further noteworthy point is that the EMG effects are not detected in the easier Academic condition even though RT compatibility effects are detected. This is clearly not a result we would have predicted. One possibility is that EMG effects are detected in the harder Sporty condition because the task is more effortful, where RTs are significantly longer, and where observation of Sporty activity might increase arousal levels (an idea suggested by a reviewer). That is, ambient increases in emotion/arousal are necessary for EMG to detect the more subtle discrimination of compatible and incompatible trials. We have recently noted that the ability of EMG to discriminate between different conditions, such as mimicking smiling vs. frowning emotions, can be affected by prior general conditions that might increase negative mood states, such as emotion inconsistency (Kirkham, Hayes, Pawling, & Tipper, under review).

In sum, our serendipitous findings raise interesting issues in spatial interference tasks similar to the Simon effect. We confirm that robust spatial interference effects are observed via RTs in some conditions but not others. However, we discovered that EMG can discriminate compatible from incompatible conditions long after a response has been evoked and when there were no effects in RTs. The theoretical implications of this finding need to be resolved, but they emphasize the importance of employing diverging methods to study basic processes such as response competition and selection for action (see Manssuer et al., in press). The majority of research in experimental psychology has traditionally developed theory based on behavioural measures such as RTs and error rate, but other simultaneous measures may tell a different story.
